# An enhanced version of the PHIRI infrastructure: improving the technological solutions

**DOI:** 10.1093/eurpub/ckac129.469

**Published:** 2022-10-25

**Authors:** P Derycke

**Affiliations:** Sciensano, Brussels, Belgium

## Abstract

The proof of concept tested by PHIRI consisted of the development of several research questions in multiple data hubs using a federated approach. It was possible to embed the use cases’ analytical pipelines in a portable standalone (i.e. docker image) and distribute it in different health data hubs and technological environments sources for execution. The tested solution has the advantage of not moving sensitive data out of the silos and thus protecting privacy - the code meets data and not the opposite. Some precious lessons provide guidance on how to further develop the PHIRI infrastructure. 1) A deep knowledge on what data is available in the different data hubs of a federation is key since the basis for the development of a research query is the construction of a data model that is common to all the nodes in the federation. In an eventual enhanced PHIRI infrastructure, a solution will be implementing a semantic information system that allows the exchange of metadata using federated and interoperable metadata catalogues based on Semantic RDF graph databases, compliant with the W3C DCAT metadata standard and exposing the end-points of the SPARQL querying language of the Web of linked-data. 2) Making available training samples mimicking real-world data within the docker image has been of high added-value for the development of the use cases’ analytical pipelines. In an eventual enhanced PHIRI infrastructure, a generalisation could consist of setting up a “knowledge hub” where synthetic data, twinning the population, data would allow any expert users to search and find data through federated queries and prepare and train their analytical pipelines; the “knowledge hub” would provide a computational environment (e.g. Jupyter as a service playground), the necessary tools (i.e. cookbooks and capacity building services) and training samples to answer research questions, with the advantage of using data that is anonymous by nature and open access.

